# Topical Fungal Infection Induces Shifts in the Gut Microbiota Structure of Brown Planthopper, *Nilaparvata lugens* (Homoptera: Delphacidae)

**DOI:** 10.3390/insects13060528

**Published:** 2022-06-08

**Authors:** Zhengliang Wang, Yiqing Cheng, Yandan Wang, Xiaoping Yu

**Affiliations:** Zhejiang Provincial Key Laboratory of Biometrology and Inspection and Quarantine, College of Life Sciences, China Jiliang University, Hangzhou 310018, China; zhengliang.w0234@163.com (Z.W.); chengyiqing11@163.com (Y.C.); wydxcjlu@163.com (Y.W.)

**Keywords:** brown planthopper, gut microbiota, fungal entomopathogen, gut immunity

## Abstract

**Simple Summary:**

Fungal entomopathogens are important natural enemies of insect pests and widely applied for biocontrol. Gut microbiota play important roles in mediating insect physiology and behavior. There is growing evidence that alteration of gut microbial communities due to pathological and environmental exposure can have detrimental impacts on host health and pathogen resistance. Here, we investigated the effects of topical infection with *Metarhizium anisopliae* fungus on the gut microbial community structure of the brown planthopper (*Nilaparvata lugens*, BPH), a destructive insect pest of rice. Our results demonstrated dramatic changes of gut bacterial community structure after topical fungal infection in BPH, as indicated by a significant increase in bacterial load, a significant decrease in bacterial community evenness and significant shifts in dominant bacterial abundance at the taxonomic level below the class. The dysbiosis of the gut bacteria might partly be due to the suppression of gut immunity caused by topical fungal infection. Our results highlighted the importance of the gut microbial community in fungal pathogenesis in insects.

**Abstract:**

The brown planthopper (*Nilaparvata lugens*, BPH) is a destructive insect pest posing a serious threat to rice production. The fungal entomopathogen *Metarhizium anisopliae* is a promising alternative that can be used for BPH biocontrol. Recent studies have highlighted the significant involvement of gut microbiota in the insect–fungus interactions. In the presented study, we investigated the effects of topical fungal infection on the gut microbial community structure in BPH. Our results revealed that topical infection with *M. anisopliae* increased the bacterial load and altered the bacterial community structure in the gut of BPH. The relative abundances of the dominant gut bacteria at the order, family and genus level were significantly different between fungus-infected and uninfected groups. At the genus level, the uninfected BPH harbored high proportions of *Pantoea* and *Enterobacter* in the gut, whereas the fungus-infected BPH gut was absolutely dominated by *Acinetobacter*. Moreover, topical fungal infection significantly inhibited the expressions of immune-related genes encoding anti-microbial protein and dual oxidase that were involved in the maintenance of gut microbiota homeostasis, indicating that gut bacteria imbalance might be attributed in part to the suppression of gut immunity caused by fungal pathogen. Our results highlighted the importance of the gut microbial community during interactions between fungal pathogens and insect hosts.

## 1. Introduction

The brown planthopper (BPH), *Nilaparvata lugens* Stål (Hemiptera: Delphacidae), is one of the most destructive piercing-sucking pests on rice (*Oryza sativa* L.). It can cause damages directly through sucking sap from the phloem of rice plants and indirectly via transmitting plant-pathogenic viruses, resulting in substantial yield and economic losses every year in rice producing areas [1]. Chemical control is the primary method for controlling this pest. However, BPH has evolved heavy resistance to a variety of conventional chemical insecticides [2]. Additionally, the long-term and unreasonable using of synthetic chemicals has also caused severe environmental pollution and ecological damage [2]. Thus, environmentally friendly alternative methods for BPH control are urgently required.

Numerous practical research evidenced that an effective alternative approach for BPH control is to make use of fungal entomopathogens [3,4,5,6,7]. Entomopathogenic fungi infect their host insects via attachment of conidia to the host cuticle, then invade into host body by conidia germination and hyphal penetration, and finally proliferate in insect haemocoel and kill the hosts [8]. At present, many fungal strains with high virulence to BPH have been screened based on laboratory and/or filed bioassays, and most of them belonging to *Beauveria*, *Metarhizium* and *Isaria* [5,6,7]. For example, *Metarhizium anisopliae* CQMa421 has shown high control efficiency against BPH during both the nymphal and adult stages, but without adverse effects on natural enemies [7]. Nevertheless, fungal pesticidal agents suffer the disadvantage of having a relatively slower killing speed when compared with chemical insecticides, which hampered their widespread application [9]. To develop approaches to enhance fungal pesticidal efficacy, a better understanding of the insect–fungus interaction is required.

Fungal infection often triggers the insect innate immune system, including cellular and humoral responses [8]. In the insects–fungi interaction model, immune aspects in insects have been extensively studied in individuals or tissues (hemolymph and fat body) that are traditionally attributed to immune responses [10,11,12]. A large number of immune-related genes have been found to be up-regulated in the whole BPH body after topical infection with *M. anisopliae* based on comparative transcriptomic analysis [13,14]. Nowadays, accumulating studies have revealed that the gut also plays an important role in shaping insect immunity.

The insect gut is a complex ecosystem consisting of diverse communities of microbes that play important roles in host physiology including nutrition metabolism, immunity modulation and pathogen defense [15]. Recent studies have shown that insect gut microbiota could affect to the pathogenic process and the pesticidal efficiency of insect pathogens [16,17,18,19,20,21]. For example, gut microbiota of the beet armyworm *Spodoptera exigua* could enhance baculovirus virulence by modulating gut immunity [16]. Similar effects were found for the cotton bollworm *Helicoverpa armigera* [17], the gypsy moth *Lymantria dispar* [18] and the malaria mosquito *Anopheles stephensi* [19] in response to viral, bacterial and fungal challenged, respectively. In contrast, gut bacteria in the honey bee *Apis mellifera* [20] and cockroach *Blattella germanica* [21] could protect their hosts against invading pathogens by up-regulated the host immune response or by producing antimicrobial compounds. To date, the interaction between insect gut microbiota and pathogen infection was mainly based on models of “insect-virus” or “insect-bacteria” [16,17,18,20]. However, unlike viral and bacterial pathogens, which invade insects through their oral cavity and/or gut, insect fungal pathogens infect insects primarily through the cuticle [8]. Hence, the interactions between insect gut microbiota and fungi might be more complex and fascinating. However, few reports have explored the interplay between fungal infection and insect gut microbial associates.

In this study, we aim to investigate how gut microbitoa in BPH respond to topical infection with a fungal entomopathogen, *M. anisopliae*, which has great potential for BPH biocontrol [3,14]. Building on the literature and preliminary research, we hypothesized that the gut microbial community homeostasis would be disturbed after fungal infection by topical route in BPH. To test this hypothesis, we determined the changes in the composition of gut bacterial community after topical fungal infection by in vitro culture, quantitative polymerase chain reaction (qPCR) and high-throughput 16S rRNA amplicon sequencing. Moreover, the expression patterns of gut-homeostasis-related genes in the gut of BPH during the course of fungal infection were assessed by quantitative real time polymerase chain reaction (qRT-PCR) analysis.

## 2. Materials and Methods

### 2.1. Insect and Fungal Entomopathogen

The BPH population was originally collected from rice fields in a paddy field in Yuyao, Zhejiang province of China (121°33 E, 29°99 N) and maintained on the rice variety TN1 in the insectary greenhouse for more than 20 generations under controlled conditions (27 ± 1 °C, 70 ± 10% relative humidity and a 14:10 h light/dark photoperiod). The entomopathogenic fungal strain *M. anisopliae* ARSEF456 (designated as Ma456 herein) was grown on the plates of Potato dextrose agar (PDA) at the regime of 28 °C and 12:12 h (light/dark cycle).

### 2.2. Topical Fungal Infection and Gut Dissection

Aerial conidia of Ma456 produced on PDA plates were washed with 0.02% Tween 80 solution and adjusted to a final concentration of 1 × 10^8^ conidia/mL using a hemocytometer. BPH nymphs (24 h after molting at the fifth instar) were prepared for topical fungal infection following previous protocol [14]. Briefly, batches of 30–40 nymphs on 3-cm high rice seedlings in uncaged cups were sprayed with 1 mL conidial suspension using a handheld micro sprayer. The amount of conidia deposited onto the nymphs was measured as number of conidia mm^−2^ using microscopic counts of conidia collected onto four glass slips (20 × 20 mm) under each spray. Control nymphs (CK group) were treated with an equal-volume of 0.02% Tween 80. In order to test whether 0.02% Tween 80 has detrimental impacts on the gut microbial community structure, BPH nymphs were also sprayed with 1 mL ddH_2_O. All sprayed nymphs were reared in situ in a growth chamber at 25 °C and a 14:10 h light/dark photoperiod and BPH mortality was recorded daily for 10 days. Fresh rice seedlings were supplied every 3 days for their feeding during the period of rearing.

For sampling of BPH gut, samples of 400 surviving nymphs after post-infection of 4 days were collected from the fungal treatment group and the control group, respectively. Prior to gut dissection, all nymphs were surface sterilized by washing them three rinses with 75% ethanol for 1 min each time, followed by three rinses with ddH_2_O for 1 min each time. Gut samples were gently dissected by using sterile forceps under a stereomicroscope and then homogenized in 1 mL phosphate-buffered saline (1× PBS) and frozen at −80 °C. All gut samples were divided into four sets for in vitro microbial culture, DNA and RNA extraction, respectively.

### 2.3. Quantification of Gut Bacteria by CFU Counting Assay

To check the quantity changes of BPH gut microbiota in response to fungal infection, gut samples suspended in 1× PBS were diluted to a suitable concentration (10^−2^–10^−5^) and 100 μL aliquots of suspension of each gut sample were spread onto the surface of Luria-Bertani (LB) agar plates, followed by 24–48 h incubation at 37 °C. Then, the colony forming units (CFUs) of gut bacteria were counted and calculated for each gut. At least five replicates for each sample were used for analysis.

### 2.4. Quantification of Gut Bacteria by 16S rRNA Gene qPCR Assay

Total DNA of BPH gut was extracted using the DNeasy Tissue Kit (Qiagen, Hilden, Germany) according to the manufacturer’s protocol. The quality and quantity of the extracted DNA were determined by a Nanodrop 2000 spectrophotometer (Thermo Fisher Scientific, Waltham, MA, USA) and 1% agarose gel electrophoresis. The total gut bacterial load was quantified by qPCR with a pair of universal primers 1114F (5′-CGGCAACGAGCGCAACCC-3′) and 1275R (5′-CCATTGTAGCACGTGTGTAGCC-3′) targeting 16S rRNA gene. All qPCR reactions were carried out in triplicate with SYBR^®^ Premix Ex TaqTM (Takara, Kusatsu, Japan) according to the manufacturer’s instructions. The BPH housekeeping 18S ribosomal protein gene (18S) was used as the internal standard.

### 2.5. DNA Extraction, PCR Amplification and High-Throughput Sequencing

Total gut DNA from Ma456-infected and 0.02% Tween 80-treated nymphs was extracted and qualified in preparation for 16S rRNA gene amplicon sequencing as the method described as above. Bacterial V3-V4 region of the 16S rRNA gene was amplified using specific barcoded primers 338F (5′-barcode-ACTCCTACGGGAGGCAGCAG-3′) and 806R (5′-barcode-GGACTACHVGGGTWTCTAAT-3′). The barcode fragments were used to sort multiple samples in a single sequencing run. PCR reactions were performed in a total volume of 25 µL, containing 2.5 μL 5× FastPfu Buffer, 2 μL dNTPs (2.5 mM), 0.5 μL each primer (10 μM), 0.5 μL FastPfu Polymerase (5 U/μL), 1 μL template DNA (about 50 ng) and 13 μL ddH_2_O. The PCR procedures were as follows: an initial denaturation for 3 min at 94 °C, followed by 30 cycles of denaturation for 30 s at 94 °C, annealing for 30 s at 55 °C, elongation for 30 s at 72 °C, and a final extension step for 5 min at 72 °C. PCR reactions were conducted in triplicate for each sample and the PCR products were pooled to minimize the PCR bias. After evaluation by 2% agarose gel electrophoresis, the high-quality amplicons from each sample were adjusted to an equal concentration and subsequently sent for sequencing on an Illumina NovaSeq platform according to the standard protocols at LC-Bio Technology (Hangzhou, China). Each experiment was repeated with three independently isolated DNA samples (biological replicates).

### 2.6. 16S rRNA Gene Amplicon Sequence Analysis

Paired-end reads were assigned to appropriate samples based on unique barcodes and truncated by cutting off the primer and barcode sequence, and then assembled using FLASH software (Columbia, MD, USA) [22]. Raw sequences were quality-filtered using QIIME version 1.8.0 [23]. Low complexity sequences, sequences with ambiguous bases and sequences with length below 250 bp were discarded. Operational units (OTUs) were clustered with a 97% similarity cut-off using UPARSE version 7.1 [24]. The taxonomic classification of each bacterial OTU was assigned by RDP Classifier (http://rdp.cme.msu.edu/, accessed on 20 February 2022) against the Silva (SSU115) 16S rRNA database using a confidence threshold of 70%. The alpha diversity and beta diversity indices were calculated using QIIME V1.8.0. Alpha diversity analysis is used to analyze complexity of species diversity for a sample, including Chao1 index, Simpson index and Shannon estimator. Beta diversity was applied to evaluate structural variation of bacterial community among samples using the weighted UniFrac distance metric, and visualized using principal coordinate analysis (PCoA). Linear discriminant analysis coupled with effect size measurements (LEfSe) was applied to identify differentially abundant bacterial taxa among groups. Only those taxa that obtained a log linear discriminant analysis (LDA) score > 3.0 and *p*-value < 0.05 were ultimately considered. The raw data have been deposited into NCBI Sequence Read Archive database under the accession number PRJNA832103.

### 2.7. qRT-PCR Analysis of Gut-Homeostasis-Related Genes

Total gut RNA from Ma456-infected and 0.02% Tween 80-treated nymphs was extracted using TRIzol^®^ Reagent and treated with DNase I (New England Biolabs, Ipswich, UK) according to the manufacturer’s instructions. After evaluation by RNase-free agarose gel electrophoresis and a NanoDrop 2000 spectrophotometer, all RNA samples were reversely transcribed into cDNAs with PrimeScriptTM RT kit (Takara, Kusatsu, Japan) and then assessed for the transcript levels of ten gut-homeostasis-related genes via qRT-PCR with paired primers (Table S1). All qRT-PCR experiments were performed with SYBR^®^ Premix Ex Taq^TM^ (Takara, Kusatsu, Japan) under the following conditions: an initial denaturation for 30 s at 95 °C, followed by 40 cycles of 5 s at 95 °C and 30 s at 60 °C, and a final step for generation of melting curves. The 18S ribosomal protein gene (18S) of BPH was used as the internal standard. The relative expression level of each gene in each group was estimated using the 2^−ΔΔCt^ method [25]. qRT-PCR analysis was conducted in the triplicate assays, each of which contained three technical replicates.

### 2.8. Statistical Analysis

Statistical analyses were performed using DPS software v7.05 [26]. Differences between the fungal treatment group and control group in bacterial CFU counts, bacterial *16S rRNA* gene quantification, alpha diversity, bacterial taxa abundance at different taxonomic levels and immune-related gene expression level were analyzed by unpaired two-tailed Student’s *t*-test or one way analysis of variance (ANOVA), followed by Tukey’s honestly significant difference (HSD) test. Differences were considered significant if *p*-value < 0.05.

## 3. Results

### 3.1. Fungal Infection Caused High Mortality of BPH Nymphs

**Figure 1:**
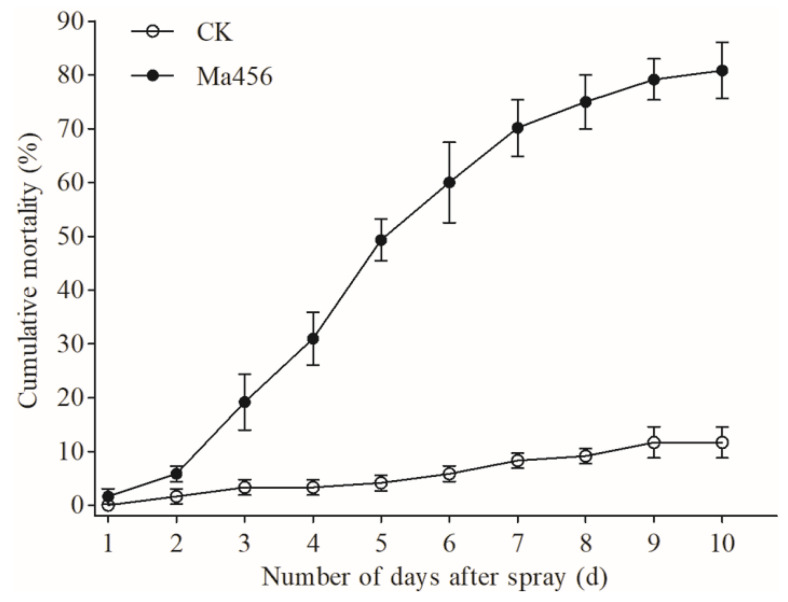
Cumulative mortality rate of BPH fifth-instar nymphs infected by Ma456. Error bars: SD of the mean from three replicates.

A laboratory bioassay was conducted to verity the virulence of the fungal conidia to the and to fifth-instar nymphs of BPH. The concentrations of Ma456 conidia deposited onto BPH nymphs were 952 ± 84 conidia mm^−2^ and showed no statistically significant difference among the bioassay replicates. Cumulative mortality of BPH nymphs during a 10-day observation period after exposure to fungal spray was illustrated in Figure 1. Corrected mortality of 27.7% and 54.2% for sprayed nymphs were observed at 4 and 6 days post infection (dpi), respectively, and reached 69.2% at 10 dpi.

### 3.2. Fungal Infection Enhanced Bacterial Load in BPH Gut

**Figure 2:**
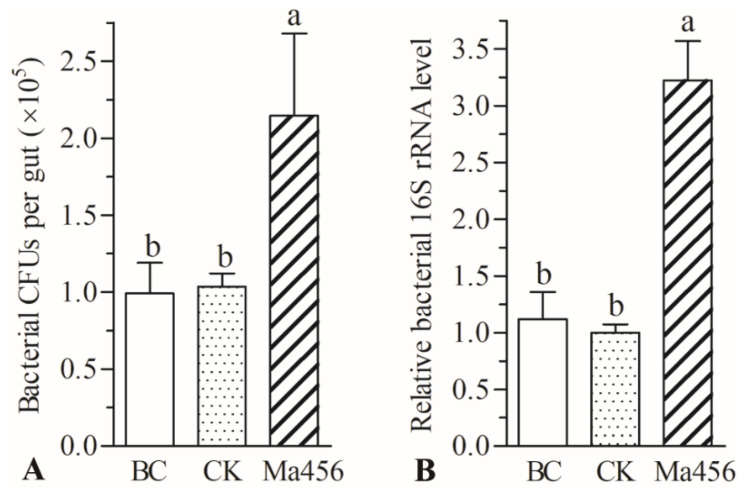
Bacterial CFUs count (**A**) and relative quantification of bacterial 16S rRNA (**B**) in the gut of BPH treated by topical spraying fungal conidia (Ma456), 0.02% Tween 80 (CK) and ddH_2_O (BC), respectively. Different letters on the bars of each group denote significant differences among BPH gut samples (*p* < 0.05). Error bars: SD from three repeated assays.

The cultivable bacteria loads in the gut of BPH at 4 dpi were determined by the CFU counting assays. As a result, the load of the culturable bacteria was significantly increased in topical fungal infected groups compared with uninfected controls. The number of bacterial CFUs in the gut of BPH at 4 dpi was 2.15 ± 0.53 × 10^4^ per gut, which were 2.1-fold higher than that in the 0.02% Tween 80-treated group, in which 1.04 ± 0.09 × 10^4^ CFUs per gut was detected (Figure 2A). The qPCR result also showed that the total bacterial load in the fungus-infected group was significantly higher (about 3.2-fold) than that in the control groups (Figure 2B). No significant differences in both bacterial CFUs count and relative bacterial 16S rRNA level were observed between 0.02% Tween 80-treated and ddH_2_O-treated groups.

### 3.3. Fungal Infection Decreased Bacterial Community Evenness in BPH Gut

16S rRNA gene amplicon sequencing generated a total number of 494,588 raw reads from six gut samples. After quality filtering and chimera removal, a total of 445,848 clean reads were remained, including 187,059 reads from the fungus-infected group and 258,789 reads from the control group, which then clustered into a total of 192 and 169 bacterial OTUs at a 97% similarity level, respectively (Table 1). The taxonomy of all gut bacterial OTUs was presented in Table S2 (online only). The alpha diversity indices were estimated using three measurements, including Chao1, Simpson and Shannon indices. As a result, there were no significant differences between the fungus-infected BPH and the control BPH in terms of Chao1 index that reflected microbial community richness. However, the fungus-infected group shown a markedly lower Simpson and Shannon diversity indices when compared with the control group (*p* < 0.05), indicating that topical fungal infection decreased the bacterial community evenness (Table 1).

### 3.4. Fungal Infection Altered Bacterial Community Composition in BPH Gut

**Figure 3:**
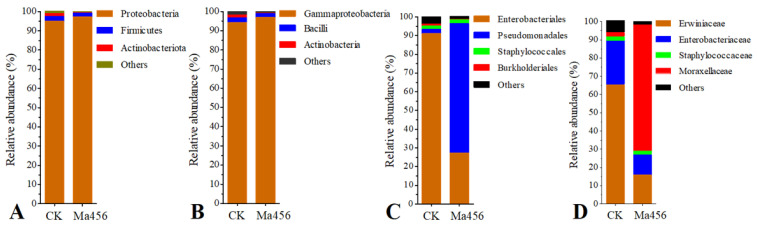
The bacteria with the relative abundance over 1.00% in at least one BPH population at the levels of phylum (**A**), class (**B**), order (**C**) and family (**D**) in the gut of BPH that treated by fungal infection (Ma456) and 0.02% Tween 80 (CK), respectively.

**Figure 4:**
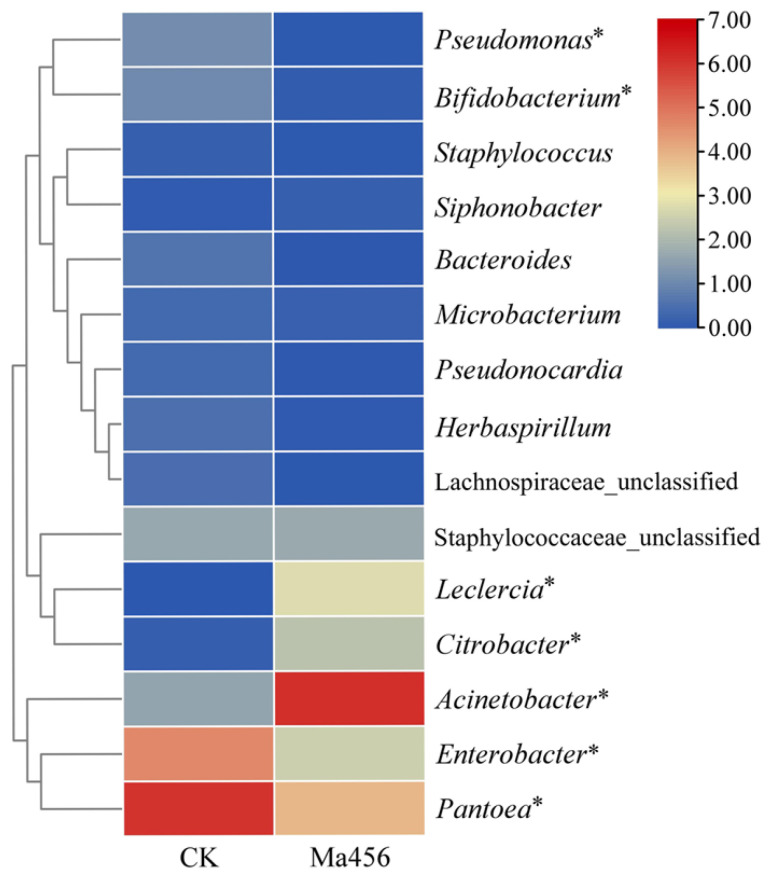
Heatmap of the relative abundance of the top 15 predominant bacterial genera in the gut of BPH that were treated by fungal infection (Ma456) and 0.02% Tween 80 (CK). The color scale represents values of relative abundances (%) normalized by log_2_. Zero values were added as 1 and log_2_ transformed. Asterisked species differ significantly in the relative abundance between two BPH gut samples (*p* < 0.05).

Based on the OTU classification, a total of 13 bacterial phyla consisting of 18 classes, 44 orders, 69 families and 111 genera were assigned in the fungus-infected gut samples, while the total bacterial OTUs in the control gut samples were annotated into 11 phyla, 17 classes, 43 orders, 61 families and 101 genera. The bacteria with the relative abundance over 1.00% in at least one group at the levels of phylum, class and order in BPH gut were shown in Figure 3. No significant differences were observed in the relative abundance of bacteria at the level of phylum and class. The most dominant gut bacteria in both BPH groups belonged to the phylum Proteobacteria (Figure 3A) and the class Gammaproteobacteria (Figure 3B), accounting for more than 90% in each group. At the order level, the bacteria from Pseudomonadales were the most predominant in the fungus-infected gut samples. However, the most dominant bacteria in the control gut samples were represented by Enterobacterales (Figure 3C). The relative abundance of Pseudomonadales in the fungus-infected group was 69.16 ± 8.47%, which was significant higher than that in the control group (2.37 ± 1.08%). By contrast, the relative abundance of Enterobacterales in the fungus-infected group (27.30 ± 9.36%) was significant lower than that in the control group (90.98 ± 7.00%). Among the bacterial families, Moraxellaceae was the most dominant family in the fungus-infected gut samples (69.15 ± 8.47%), followed by Erwiniaceae (15.88 ± 8.73%) and Enterobacteriaceae (11.24 ± 3.61%). However, significant lower abundance of Moraxellaceae (2.19 ± 0.69%), and significant higher abundance of Erwiniaceae (65.43 ± 4.37%) and Enterobacteriaceae (23.85 ± 7.38%) were observed in the control gut samples (Figure 3D). The variations in bacterial community compositions at the genus level were visualized on the heat map of top 15 abundant bacteria (Figure 4). Remarkably, bacterial communities from the fungus-infected gut samples were dominated by members of the genus *Acinetobacter*, with the relative abundance of 68.48 ± 7.32%, which was extremely higher than that in the control gut samples (about 2%). In contrast, the dominant genus of bacteria in the control gut samples was represented by *Pantoea* and *Enterobacter*, with the relative abundance of 64.76 ± 3.50% and 25.21 ± 8.42%, respectively.

**Figure 5:**
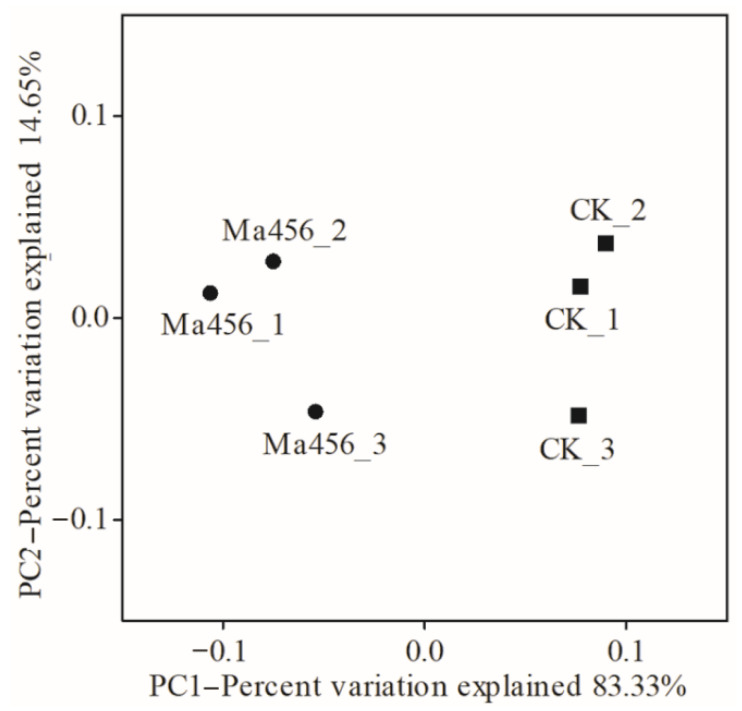
Principal coordinate analysis (PCoA) of beta diversity based on the weighted UniFrac distance metric for gut bacterial communities in the fungus-infected (Ma456) and 0.02% Tween 80-treated (CK) BPH.

To more rigorously compare the bacterial community structure between the fungus-infected and control group, a PCoA analysis plot of samples using the weight UniFrac distance metric was performed. As shown in Figure 5, the bacterial communities from the fungus-infected gut samples clustered independently and distinctly from the control gut samples based on the weighted UniFrac PCoA plot, as significant differences were observed between them along the PC1 axis (*p* < 0.05). Based on this analysis the BPH gut bacterial community following exposure to Ma456 was distinct from untreated guts. The observed differences in beta-diversity were directly reflected by the strong shifts in the taxonomic composition of the gut bacterial community in BPH after fungal infection that was described above. For instance, the relative abundance of Moraxellaceae was noticeably higher in the fungus-infected group, whereas Erwiniaceae were significantly enriched in the control group. At the genus level, more than one-third of the bacterial genera had significant differences in the relative abundance between the fungus-infected and control group, such as *Pantoea*, *Acinetobacter*, *Enterobacter*, *Leclercia*, *Citrobacter* and *Pseudomonas* (Figure 3).

**Figure 6:**
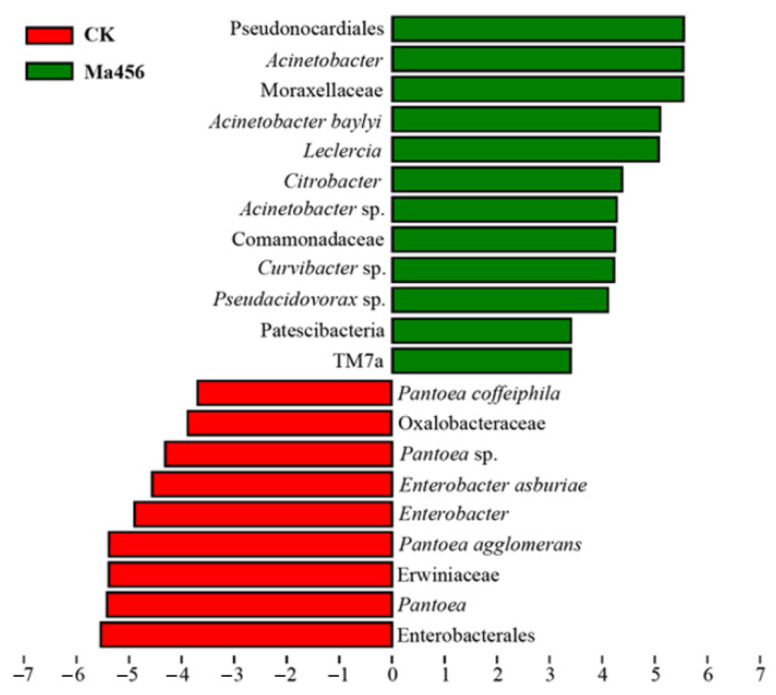
Different structures of gut bacteria between the fungus-infected (Ma456) and 0.02% Tween 80-treated (CK) groups identified by LEfSe analysis with linear discriminant analysis (LDA) scores of 3.0. Differentially abundant taxa are represented by histograms with LDA scores (red, CK; green, Ma456).

LEfSe analysis was also applied to identify gut bacterial taxa that differed significantly in abundance between the fungus-infected and control group. A total of 21 differentially abundant taxa were detected between the two groups, all of which had a log LDA score > 3.0 (Figure 6). For instance, at the genus level, bacteria from the fungus-infected group were enriched with *Acinetobacter* from family Moraxellaceae, *Leclercia* and *Citrobacter* from family Enterobacteriaceae, while the relative abundances of the bacterial taxa from *Pantoea* belonging to family Erwiniaceae and Enterobacter belonging to family Enterobacteriaceae were higher in the control group. The LEfSe analysis was consistent with the results from the comparative analysis of bacterial composition community as presented above.

### 3.5. Fungal Infection Modulated Expressions of Gut-Homeostasis-Related Genes in BPH

**Figure 7:**
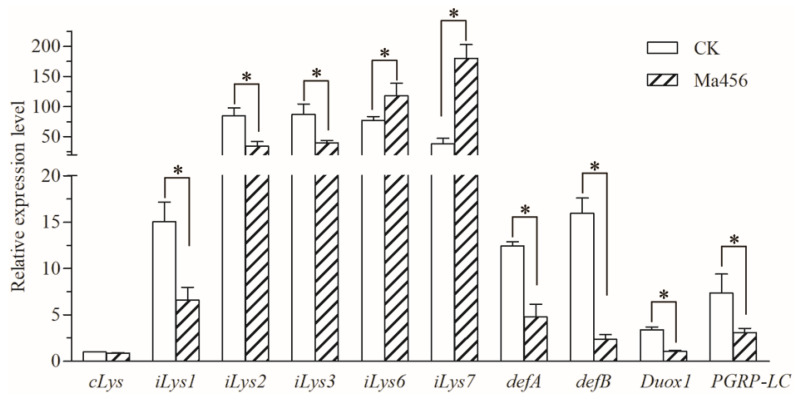
Relative expression levels of ten gut-homeostasis-related genes in the gut of BPH that treated by fungal infection (Ma456) and 0.02% Tween 80 (CK). Asterisk indicates significant difference between two BPH gut samples (*p* < 0.05). Error bars: SD from three repeated assays.

Ten gut-homeostasis-related genes were assessed for the transcript levels in the gut of BPH after topical fungal infection through qRT-PCR. As illustrated in Figure 7, nine gut-homeostasis-related genes were differentially expressed in the fungus-infected gut samples when compared to the control. Among them, two antimicrobial peptide (AMP) encoding genes (*defA* and *defB*) and three immune responsive effector genes encoding i-type lyzysome (*iLys1*, *iLys2* and *iLys3*) were significantly down-regulated their transcript levels after fungal infection. For instance, the transcript levels of *defA* and *defB* were significantly repressed by 61.4% and 85.3% in the gut of fungal-challenged BPH, respectively. A pattern recognition receptor gene encoding peptidoglycan recognition protein LC (*PGRP-LC*) involved in the immune deficiency (Imd) signal pathway and a dual oxidase (DUOX) encoding gene (*Duox1*) linked to the production of reactive oxygen species (ROS) were also significantly down-regulated their transcript levels in the gut of BPH when suffering fungal infection. However, two immune responsive effector genes encoding i-type lyzysomes (*iLys6* and *iLys7*) were significantly up-regulated their transcript levels in the fungus-infected gut samples relative to the control.

## 4. Discussion

Insect gut microbiota play vital roles in host ecology and physiology, particular in provision of nutrition necessary that essential for host growth and modulation of host immune defense against their pathogens [10,27,28]. Many factors, including the developmental stage, sex and phylogeny of the host, stressful condition and environmental habitat, have been shown to affect gut microbial community structure in insects [29]. Recently, the effects of insecticide and host rice varieties on the gut bacterial composition of BPH were assessed by high-throughput amplicon sequencing [30,31,32]. However, the influence of pathogen infection on the gut microbial composition and structure of BPH is still unclear. To fill this gap, we characterized and compared the gut bacterial community in BPH sprayed with and without the conidia suspension of an insect fungal pathogen.

Numerous studies have confirmed that *per os* infection by microbial pathogens (e.g., bacteria and virus) could influence the gut microbial community structure in insects, and the changes of gut microbial composition could in turn to alter host susceptibility to pathogen infection [16,17,18]. Insect fungal pathogen attack and kill insects primarily by penetrating the host integument and proliferating in the hemocoel cavity by exhausting host nutrients and producing toxins, *per os* infection occurs occasionally or even rarely [8]. In view of this, previous studies focusing on the interactions between fungal pathogens and host insects paid little attention to the status of gut microbiota, especially when the host was a homopteran insect with piercing and sucking mouthparts. Our results revealed that topically fungal infection could also cause a dramatic alteration in gut bacterial community structure in the sap-sucking homopteran rice pests BPH, as indicated by a significant increase in gut bacterial load and a significant decrease in bacterial community evenness. However, the changes in bacterial load and evenness did affect the status of the dominant gut bacterial phylum. According to the bacterial OTUs classification analysis, bacteria affiliated with the phylum Proteobacteria was found to be the most predominant in both fungus-infected and control gut samples, in consistent with the data reported in previous studies on the gut microbiota of diverse insect groups, including data for BPH populations with different virulence levels and insecticide-resistant levels [29,30,31,32]. This seems to imply that Proteobacteria are widely present in the gut in insects and play a vital role in host fitness and environmental adaptability.

Although the most dominant gut bacteria at higher taxonomic levels (phylum and class) showed no significant differences in BPH after fungal challenge, the relative abundances of the dominant order, family and genus of gut bacterial community were significantly different. Remarkably, at the genus level, the bacteria from *Pantoea* were the most prevalent in the control gut samples, whereas the fungus-infected gut samples was absolutely dominated by *Acinetobacter* (about 70%). *Acinetobacter* is a bacterial genus commonly found in the insect gut samples, including species of a symbiotic nature which could provide their hosts with essential nutrients [33], and species with insecticidal potential that could serve as a pathogen against their hosts [34]. The shift in dominant bacteria from *Pantoea* to *Acinetobacter* in the gut of BPH highly suggested that *Acinetobacter* might play an important role in the course of fungal infection. Recently, a significant increase in the abundance of *Serratia* and *Erwinia* was also observed in the guts of *Anopheles* mosquitos and *Dendroctonus* beetles after topical fungal infection, respectively, and furthermore, these gut bacteria overgrown in the gut reciprocally could promote the killing speed of fungal pathogen against their host insects [19,35]. Whether *Acinetobacter* have the ability to promote the fungal killing of BPH is still unclear and warrants further investigation.

Accumulating studies have proved that homeostasis in the gut bacterial community is partly determined by gut immunity [36,37,38]. The Imd signal pathway regulating the production of AMPs and the DUOX-ROS system leading to the production of ROS are considered to be the two major pathways for insects to maintain the homeostasis of gut microbiota [39,40,41]. In this study, a pattern recognition receptor encoding gene (*PGRP-LC*) and two AMP encoding genes (*defA* and *defB*) involved in the Imd pathway and a DUOX encoding gene (*Duox1*) involved in DUOX-ROS system were observed significantly repressed in the gut of BPH when suffering Ma456 challenge, suggesting that topical fungal infection could cause a level of immune suppression in the gut of BPH, which might subsequently lead to dysbiosis of its gut bacterial community. Recent studies have also shown that the suppression of immune responses by fungal invasion was attributed to the shifts of bacterial community structure in the insect gut [19,42,43]. For instance, the expressions of one DUOX and five AMPs encoding genes in the midgut of *A. stephensi* were significantly down-regulated after topical infection with an insect fungal pathogen *Beauveria bassiana*, which subsequently cause a significant increase in gut bacterial load and a significant decrease in bacterial diversity [19]. A similar phenomenon was also observed in the Colorado potato beetle when topically infected by *M. robertsii*, in which a gene regulating the activity of the DUOX was significantly inhibited along with the proliferation of *Serratia* in the gut during fungal infection [42]. Gut microbiota imbalance might also be caused by host physiological reactions under stressful conditions, including pathogen infection and insecticide exposure [44,45]. The micro-ecology of the insect gut might be altered by toxic compounds released by fungal pathogen during infection, such as oosporein secreted by *Beauveria*, which was proved to be effective in changing the gut bacterial community in mosquito via weakening the host DUOX-ROS system [19]. *M. anisopliae* also produce bioactive metabolite destruxins that are toxic to insect hosts [46]. The interaction among fungal destruxin, insect gut immunity and gut microbiota could be the subject of future studies.

In summary, we demonstrated dramatic changes in BPH gut bacterial community structure after topical fungal infection, as expressed by a significant increase in bacterial load, a significant decrease in bacterial community evenness and significant changes in dominant bacterial abundance at the taxonomic level below the class. The suppression of gut immunity might partly account for the gut microbiota imbalance. Our results highlighted the importance of considering the gut microbial community when determined the interactions between fungal pathogen and insect host.

## Figures and Tables

**Table 1:** Bacterial community alpha-diversity characteristics in the gut of BPH infected and uninfected with fungal pathogen.

Sample	Raw Tags	Valid Tags	OTUs	Shannon †	Simpson †	Chao1 †	Coverage †
CK	219,128	187,059	192	3.03 (±0.32) ^a^	0.76 (±0.01) ^a^	76.67 (±20.54) ^a^	0.999 (±0.001)
Ma456	275,460	258,789	169	1.91 (±0.41) ^b^	0.59 (±0.06) ^b^	77.11 (±7.52) ^a^	1.000 (±0.000)

† Numbers represent mean (±standard error) and different lowercase letters on the same row indicate differences for *p* < 0.05. CK and Ma456 refer to the gut sample from 0.02% Tween 80-treated group and fungus-infected group, respectively.

## Data Availability

The data presented in this study are available in the article and Supplementary Materials.

## Supplementary Materials

The following supporting information can be downloaded at: https://www.mdpi.com/article/10.3390/insects13060528/s1; Table S1: Specific primer pairs of the gut-homeostasis-related genes for qRT-PCR.; Table S2: Bacterial OTUs in the gut of BPH infected and uninfected by fungal pathogen.
