# Supplementary material for: Topical Fungal Infection Induces Shifts in the Gut Microbiota Structure of Brown Planthopper, Nilaparvata lugens (Homoptera: Delphacidae)
Source: Insects. 2022 Jun 8;13(6):528. doi: 10.3390/insects13060528 (PMC9225076; doi:10.3390/insects13060528)
Supplement: Supplementary file 1 [file insects-13-00528-s001.zip › Table S1.pdf]

Table S1. Specific primer pairs of the gut-homeostasis-related genes for qRT-PCR.

**Genes**	**Description**	**Primers**	**Sense/Antisense primer sequences (5′-3′)**
PGRP-LC	Peptidoglycan recognition protein LC	PGRP-LC-F/R	TGGTGCGAAACATCCAGGACTTC/TGCGGTAGCTGTAAATGTACCTATGAG
cLys	c-type lysozyme	cLys-F/R	TATCGACAACATCAGAAGC/TCATACACTATCACCCCAA
iLys1	i-type lysozyme 1	iLys1-F/R	GAGATGTGTGCGGTGTTTTC/AGCTGTCCCCTGTTGTGTAA
iLys2	i-type lysozyme 2	iLys2-F/R	TGAAGATAATCCCAACAG/AGAGTACCTCAAACCAAA
iLys3	i-type lysozyme 3	iLys3-F/R	AGGAGATGTCTGCGGCATC/TTGTACTGAGCGTCGAGCG
iLys6	i-type lysozyme 6	iLys6-F/R	AGTCTGAGGGAGCATACCA/TTCTTCAAACACTCGGGAA
iLys7	i-type lysozyme 7	iLys7-F/R	TTTCCTGGTTTTAGTTATCGCTA/GATTGCTGAAGATTCACACATTG
defA	Defensin A	defA -F/R	GCCTCTGTGATGGCACTGTA/TGGTTCGGAGTCACCCATTG
defB	Defensin B	defB -F/R	TGCCTCTGTAATGGCACTGTA/GGAGTCACCCATTTGCTGTTG
Duox1	Dual oxidase 1	duox1-F/R	TCGAGAACGGAACCCTGCTG/GCGAAAGAAAAGGATGCCGAA
18S rRNA	Internal standard gene	18S-F/R	GTAACCCGCTGAACCTCC/GTCCGAAGACCTCACTAAATCA
